# Chronic Systemic Inflammation Exacerbates Neurotoxicity in a Parkinson's Disease Model

**DOI:** 10.1155/2020/4807179

**Published:** 2020-01-11

**Authors:** Perla Ugalde-Muñiz, Ingrid Fetter-Pruneda, Luz Navarro, Esperanza García, Anahí Chavarría

**Affiliations:** ^1^Unidad de Investigación en Medicina Experimental, Facultad de Medicina, Universidad Nacional Autónoma de México, CP06726, Mexico City, Mexico; ^2^Centro de Ciencias de la Complejidad, Universidad Nacional Autónoma de México, CP04510, Mexico City, Mexico; ^3^Departamento de Fisiología, Facultad de Medicina, Universidad Nacional Autónoma de México, CP04510, Mexico City, Mexico; ^4^Laboratorio de Neuroinmunología, Instituto Nacional de Neurología y Neurocirugía Manuel Velasco Suárez, S.S., CP14269, Mexico City, Mexico

## Abstract

Systemic inflammation is a crucial factor for microglial activation and neuroinflammation in neurodegeneration. This work is aimed at assessing whether previous exposure to systemic inflammation potentiates neurotoxic damage by the neurotoxin 1-methyl-4-phenyl-1,2,3,6-tetrahydropyridine (MPTP) and how chronic systemic inflammation participates in the physiopathological mechanisms of Parkinson's disease. Two different models of systemic inflammation were employed to explore this hypothesis: a single administration of lipopolysaccharide (sLPS; 5 mg/kg) and chronic exposure to low doses (mLPS; 100 *μ*g/kg twice a week for three months). After three months, both groups were challenged with MPTP. With the sLPS administration, Iba1 staining increased in the striatum and substantia nigra, and the cell viability lowered in the striatum of these mice. mLPS alone had more impact on the proinflammatory profile of the brain, steadily increasing TNF*α* levels, activating microglia, reducing BDNF, cell viability, and dopamine levels, leading to a damage profile similar to the MPTP model *per se*. Interestingly, mLPS increased MAO-B activity possibly conferring susceptibility to MPTP damage. mLPS, along with MPTP administration, exacerbated the neurotoxic effect. This effect seemed to be coordinated by microglia since minocycline administration prevented brain TNF*α* increase. Coadministration of sLPS with MPTP only facilitated damage induced by MPTP without significant change in the inflammatory profile. These results indicate that chronic systemic inflammation increased susceptibility to MPTP toxic effect and is an adequate model for studying the impact of systemic inflammation in Parkinson's disease.

## 1. Introduction

Parkinson's disease (PD) is the second most common neurodegenerative disease and is characterized by a chronic progressive neuronal loss mainly in the substantia nigra, which causes a decrease in the production and availability of dopamine and manifests as a loss of movement control [1]. Despite the amount of research on this neurodegenerative disease, its origin remains unclear. Only 5-10% of cases have a genetic background [2–5], while the rest are of idiopathic origin [6], although some risk factors have been identified, such as age, environmental toxins, and infections [7, 8].

The inflammatory process, oxidative stress, and microglia activation are essential components in the pathogenesis of many neurodegenerative disorders such as PD [9]. Microglia are vital in the maintenance of immune homeostasis in the central nervous system (CNS). Nevertheless, during aging, microglia are activated, secrete inflammatory cytokines, and also promote the release of secondary inflammatory mediators such as prostaglandins and nitric oxide (NO) [10, 11]. Additionally, they facilitate the production of reactive oxygen species (ROS) through the induction or activation of NADPH oxidase and the release of NO [12, 13].

Microglia also respond and propagate inflammatory signals initiated in the periphery by producing the proinflammatory cytokines IL1*β*, IL6, and TNF*α* [14–16]. High levels of systemic TNF*α* can cross the blood-brain barrier (BBB), stimulating the microglia to secrete more TNF*α* as well as other proinflammatory factors and thus creating persistent and self-generated neuroinflammation [15]. Metabolic diseases such as obesity, hypertension, dyslipidemia, diabetes, and insulin resistance are associated with chronic systemic inflammation and a higher risk of developing neurodegenerative diseases such as Alzheimer's disease and PD [17–23]. Due to the importance of peripheral inflammatory processes in PD development [24–26], it is relevant to investigate more thoroughly the mechanisms involved.

**Figure 1:**
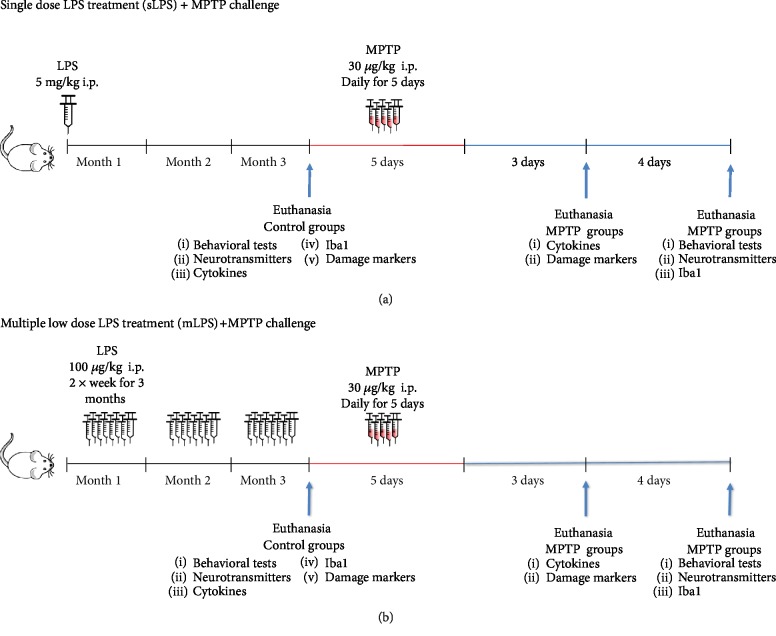
Schematic representations of systemic LPS administration regimens. For the single LPS (sLPS) treatment (a), mice were injected intraperitoneally once with 5 mg/kg. After three months, they were challenged with MPTP (30 mg/kg for five consecutive days) and were then analyzed. For the repeated LPS (mLPS) treatment (b), mice were injected intraperitoneally 100 *μ*g/kg twice a week for three months. They were then challenged with MPTP (30 mg/kg for five consecutive days) and analyzed. Control groups (saline, sLPS, and mLPS) were euthanized after three months of the challenges. MPTP groups were euthanized either three days after MPTP exposure for damage markers (MTT assay, cleaved caspase-3, and TBARS) and cytokine levels or seven days after MPTP challenge for behavioral tests, neurotransmitter levels, and Iba1 staining.

In this work, we evaluated whether systemic inflammation increases susceptibility and further damage after 1-methyl-4-phenyl-1,2,3,6-tetrahydropyridine (MPTP) exposure. For this purpose, we employed two systemic lipopolysaccharide (LPS) administration models that induce neuroinflammation, one consisting of a single high dose of LPS (5 mg/kg; Figure 1(a)) and the other of multiple low doses for three months (100 *μ*g/kg twice a week; Figure 1(b)). To test this hypothesis, we evaluated systemic inflammation through the measurement of cytokines, its impact on neuroinflammation by assessing brain cytokines, microglial response, brain NF*κ*B expression, and inflammation-induced BBB permeability. To ensure that neuroinflammation induced by peripheral LPS stimulation causes damage, we determined cell viability, cleaved caspase-3, lipid peroxidation, and neurotransmitter levels (dopamine and serotonin). Finally, we evaluated whether these phenomena are reflected in the behavior of the animals exposed to the different treatment schemes.

Both LPS models were challenged with the neurotoxin MPTP or vehicle at the end of three months of LPS treatment (Figure 1). Our results show that chronic systemic inflammation induced sustained neuroinflammation with microglia activation, TNF*α* production, BBB compromise, and cell death, inducing a parkinsonism model and conferring additional susceptibility to MPTP damage.

## 2. Methods

### 2.1. Chemicals

All reagents were of analytical grade. 3,3′,5,5′-Tetramethylbenzidine (TMB; T4444), protease inhibitor cocktail (11836153001), LPS (Lipopolysaccharides from *Escherichia coli* O111:B4; L4391), and MPTP (M0896) were obtained from Sigma Chemical Co. (St. Louis, MO, USA). Alexa Fluor 488-coupled donkey anti-rabbit (ab150073) antibody was purchased from Abcam (Cambridge, UK). Rabbit anti-Iba1 antibody (CP 290) was acquired from Biocare Medical (Pacheco, CA, USA). Human/Mouse Cleaved Caspase-3 (Asp175), DuoSet IC ELISA (DYC835), and Human/Mouse BDNF DuoSet ELISA (DY248) were purchased from R&D Systems (Minneapolis, MN, USA). 4,6-Diamidino-2-phenylindole (DAPI) antifade solution was obtained from Millipore (MA, USA). ELISA kits were bought from eBioscience (TNF*α* Mouse 88-7324; IL6 Mouse 88-7064; IL10 Mouse 88-7104; IFN*γ* Mouse 88-7314; TGF*β*-1 Human/Mouse 88-8350; and IL1*β* Mouse 88-7013).

All required solutions were prepared with deionized water from a Milli-RQ system (Millipore, MA).

### 2.2. Experimental Animals

All experiments were carried out with male CD1 (ICR) mice of 8 weeks of age, maintained under standard conditions with a 12:00 h light-dark cycle and free access to water and food. CD1 mice (ICR), as previously reported, develop a stronger proinflammatory response than C57BL/6J mice; these differences do not originate from alterations in the expression levels of TLR4 or CD14, the LPS receptors [27]. Also, CD1 mice showed depletion of the neurotransmitter dopamine and serotonin, as well as dopaminergic neuron loss in the substantia nigra, when treated with the proneurotoxin MPTP [28].

Animal handling and experimentation strictly followed the Guidelines for Care and Use of Laboratory Animals published by the National Institutes of Health and the Guidelines of the Mexican Law of Animal Protection for the use and care of laboratory animals (Norma Oficial Mexicana NOM-062-ZOO-1999). All experimental procedures were approved by the research and ethics committees of the Facultad de Medicina, Universidad Nacional Autónoma de México (Approval 043/2015). We minimized the number of mice used and their suffering or pain as much as possible.

### 2.3. LPS and MPTP Administration

Systemic inflammation was induced either with a single dose of 5 mg/kg of LPS (sLPS) administered intraperitoneally (i.p.) [15] or with multiple low doses (mLPS; 100 *μ*g/kg i.p. twice a week) for three months [29, 30] (Figure 1). Both models induce a persistent chronic neuroinflammatory state with increased brain TNF*α* levels and microglial activation in a period from three, six, up to ten months [15, 29, 30]. The control groups received saline solution twice a week for three months. After three months, sLPS and mLPS groups were challenged with MPTP (30 mg/kg i.p.) or saline solution administered daily for five consecutive days [31, 32] (Figure 1).

Animals were euthanized at two different times, depending on the type of the test to which they were assigned. The biological material used for cytokine levels and damage markers (mitochondrial function, TBARS, and cleaved caspase-3) was obtained three days post-MPTP administration; for neurotransmitter detection, Iba1, and NF*κ*B expression, this biological material was obtained seven days post-MPTP administration.

### 2.4. Motor Coordination Test with Equilibrium Bar

Motor coordination with a horizontal bar allows measuring forelimb strength and coordination [33]. For this we used an equilibrium bar of 2 mm diameter, 38 cm long, and 49 cm away from the floor. Each mouse was taken from the tail and placed quickly with the front paws at the center of the bar. The time on the bar was registered. For analysis, scores were established as follows: score 1 (1-5 seconds on the bar), 2 (6-10 s), 3 (11-20 s), 4 (21-30 s), and 5 (more than 30 s). In the case that the mouse fell from the bar, the mouse was placed again and the longest time on the bar was recorded. Lower scores show a less efficient test result. If the mouse reached the end of the column with its front paw within the 30 s, it received the score of 5. Every mouse was tested three times, and the highest score obtained from the three attempts was used for the analysis [33]. Eight to fifteen animals per group were used for statistical analysis.

### 2.5. Sucrose Consumption Preference Test

Two mice from the same experimental group were separated from the cage littermates and then housed together for the sucrose consumption preference test. Mice were evaluated for three consecutive days. Each cage contained a drinking bottle with water and one with 2% sucrose. The bottle position was changed daily to avoid preference for a place. Every day at the same time new bottles were placed, and the volume consumed in each of the previous bottles was measured. The percentage of water taken per day was calculated based on the total water consumed per cage. A percentage lower than 65% of consumption was considered anhedonia [34]. Nine to eleven animals per group were analyzed.

### 2.6. Evaluation of Dopamine and Serotonin Levels

Neurotransmitter levels were analyzed by high-performance liquid chromatography (HPLC) with electrochemical detection [32]. Briefly, animals were sacrificed by cervical dislocation, the striatum dissected and placed in microtubes with 300 *μ*L of 0.4 N perchloric acid and 0.1% of sodium metabisulfite to homogenize the tissue. Subsequently, the samples were centrifuged at 4000 × g at 4°C for 10 minutes. The supernatant was taken from each tube and stored at -80°C for further analysis on HPLC. The levels of neurotransmitters were detected in a Perkin Elmer liquid chromatograph with a BAS CC-5 electrochemical detector. An Alltech Adsorbosphere column for catecholamines (100 Å~4.6 mm) was employed. The mobile phase was composed of phosphate buffer (0.1 M, pH 3.1), 0.9 mM sodium octyl sulfate, 0.1 mM EDTA, and 15% methanol. Twenty microlitres of perchloric acid metabisulfite solution was used as a vehicle in samples and standard. All samples were analyzed by duplicate. At least five animals per group were analyzed.

### 2.7. Monoamine Oxidase B Activity Assay

The MAO-B activity was measured spectrophotometrically and is based on the formation of H_2_O_2_ from the conversion of benzylamine to benzaldehyde. The formation of H_2_O_2_ is detected by the oxidation of 3-(4,5-dimethylthiazol-2-yl)-2,5-diphenyltetrazolium bromide (MTT) in its presence. The substantia nigra of the mice was homogenized in a 1 M monosodium phosphate buffer at pH 7.2 and centrifuged at 10,000 × g. To perform the kinetics, each well of a 96-well plate contained 20 *μ*L of 1 M sodium phosphate buffer, 20 *μ*L of 50 mM benzylamine, 20 *μ*L of sample, and 40 *μ*L of water. A control well with the sample without benzylamine and a control well without sample were also included. The samples were allowed to incubate for 3 min and were placed in a BioTek plate reader to take readings every 3 minutes for 30 minutes at a wavelength of 560 nm. At least seven animals per group were used for statistical analysis.

### 2.8. BDNF and Cytokine Titration

The levels of BDNF, TNF*α*, IL6, IL10, IFN*γ*, TGF*β*, and IL1*β* were measured in serum and brain tissue by sandwich ELISA following the instructions of the provider. Mice were euthanized with sodium pentobarbital (50 mg/kg). Blood sampling was performed with a heart puncture, and after clotting, blood was centrifuged for the obtention of the serum (2500 × g, 15 min at 4°C). The whole brain was recovered and homogenized in 500 *μ*L of lysis buffer (20 mM Tris, 0.25 M sucrose, 2 mM EDTA, 10 mM EGTA, 1% Triton X-100) containing a protease inhibitor [32]. Samples, serum and brain tissue homogenates, were incubated for 18 h at 4°C with PBS-Tween 20 (0.05%)/0.5% BSA, washed three times, and incubated with the corresponding detection antibody for 2 h at room temperature. Bound detection antibodies were detected using TMB as the substrate. Optical density readings were made at 450 nm. All assays were performed by duplicate and sensitivities were 23.4 pg/mL for BDNF, 8 pg/mL for IL1*β*, 4 pg/mL for IL6, 30 pg/mL for IL10, 8 pg/mL for TNF*α*, 8 pg/mL for TGF*β*, and 15 pg/mL for IFN*γ*. At least four animals per group were used for statistical analysis.

### 2.9. Iba1 Immunofluorescence

The staining of Iba1 evaluated the microglial response in the caudal striatum and substantia nigra; both brain areas are primarily affected in Parkinson's disease [35]. For this, mice were deeply anesthetized with sodium pentobarbital (50 mg/kg i.p.) and transcardially perfused with 100 mL of phosphate-buffered saline (PBS) followed by 100 mL of a 4% paraformaldehyde solution. The brains were dissected and cryopreserved in 30% sucrose. Coronal brain sections (20 *μ*m) were obtained in a cryostat and adhered to silanized slides. Slices were permeabilized in 0.1% Triton X-100 and incubated in a 4% blocking buffer with bovine serum albumin (BSA) for 20 min at room temperature. Slices were incubated overnight with the primary antibody for Iba1 (1 : 300) at 4°C. The following day, brain slices were washed three times with PBS and incubated with the secondary antibody (1 : 500) diluted in PBS containing 2% BSA for 2 hours at room temperature, followed by three times with PBS. The slides were mounted with a DAPI antifade solution. Images were taken using a Nikon fluorescent microscope and analyzed using ImageJ software (ImageJ v1.38x; NIH, US).

The mean of fluorescence intensity was calculated using three consecutive slices from each region per animal. At least three animals per group were used for statistical analysis. The coordinates for the identification of the caudal striatum (ML = 1.5 mm, AP = 1.25 mm, and DV = 3.0 mm from Bregma) and the substantia nigra (ML = 1.5 mm, AP = −3.16 mm, and DV = 4.5 mm from Bregma) were based on the Paxinos Mouse Brain Atlas.

### 2.10. MTT Assay

3-[4,5-Dimethylthiazol-2yl]-diphenyltetrazolium bromide (MTT) reduction to formazan was measured in brain tissue homogenate as an index of the functional status of the mitochondrial respiratory chain [32, 36]. Briefly, 100 *μ*L of tissue homogenate in PBS was incubated with 10 *μ*L of MTT (5 mg/mL) for 30 minutes at 37°C protected from light. The samples were centrifuged at 15,300 × g for 3 minutes. The supernatant was removed, and the pellets were dissolved in 500 *μ*L of isopropanol. The optical density was detected at 560 nm in a plate reader BioTek (Winooski, VT, USA). Six to eleven animals per group were analyzed.

### 2.11. Determination of Cleaved Caspase-3

Cleaved caspase-3 was measured in brain homogenates by sandwich ELISA following the instructions of the provider. Samples were incubated for 18 h in a sensitized with capture antibody plate at 4°C, washed, and incubated with the detection antibody for 2 h at room temperature. Bound detection antibodies were detected using TMB as the substrate. Optical density was measured at 450 nm. All assays were made by duplicate. Four to five animals per group were used for statistical analysis.

### 2.12. Determination of Lipid Peroxidation

Lipid peroxidation was evaluated by the production of substances reactive to thiobarbituric acid (TBARS). The homogenate of the substantia nigra or the striatum was placed in a microtube with PBS and TBA reagent (0.375 g of TBA+15 g of trichloroacetic acid+2.54 mL of concentrated HCl). The samples were put in a boiling bath (94°C) for 20 min. Subsequently, the samples were centrifuged at 3000 × g for 15 min, and the optical density of the supernatant was determined with a BioTek plate reader at a wavelength of 532 nm. Six to ten animals per group were used for statistical analysis.

### 2.13. Statistical Analysis

The results are expressed as mean values ± SEM. Statistical analyses were performed using Prism (GraphPad Software 8). We used two- and one-way ANOVA to compare more than two groups of normally distributed datasets and Kruskal-Wallis test to compare more than two groups for nonnormally distributed datasets. *Post hoc* multiple comparison tests included Tukey's and Dunn's. The motor behavioral test was analyzed with the chi-square test and logistic regression. Differences were considered significant with *P* ≤ 0.05.

## 3. Results

### 3.1. Systemic LPS and MPTP Exposure Affects Behavior and Dopamine Levels

**Figure 2:**
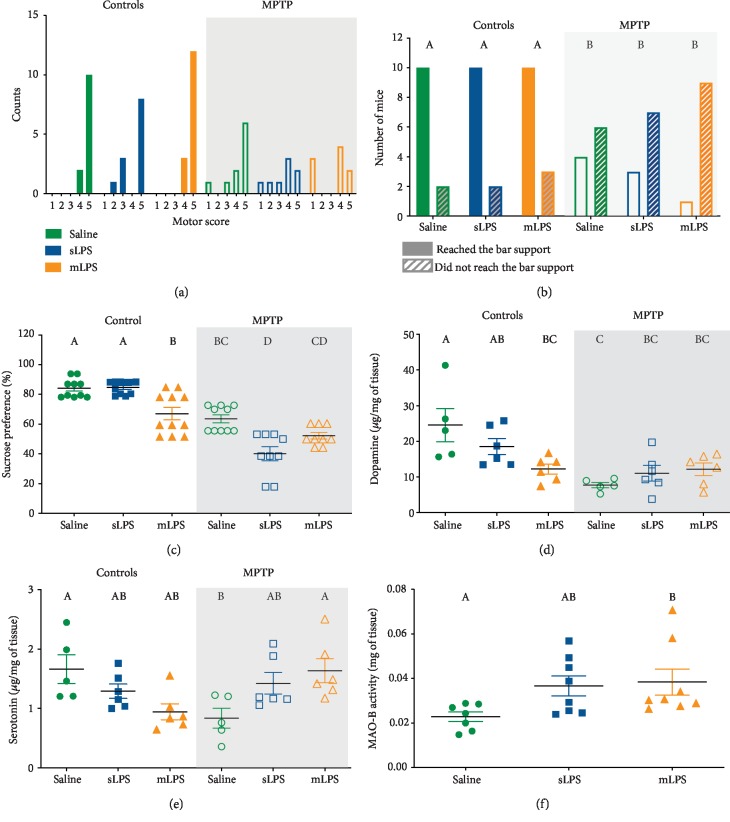
Reduction of dopamine levels relates to a decreased sucrose consumption preference, forelimb strength, and motor coordination. Mice were administered with saline solution, mLPS (100 *μ*g/kg twice a week for three months), sLPS (5 mg/kg), and MPTP (30 mg/kg for five consecutive days). Motor coordination score in mice of the different experimental groups (a). The number of mice that successfully reached the bar support (b) is indicated with colored bars. Mice that did not reach the bar support are indicated with gray stripes. Data represent mean ± SEM (*n* = 10‐12) and were analyzed by chi-square and logistic regression. Percentage of sucrose consumption preference in mice of the different experimental groups (c). Data represent mean ± SEM (*n* = 9‐11) and were analyzed by two-way ANOVA, followed by a Tukey's *post hoc* test. Different letters indicate significant differences among the experimental groups (*P* < 0.05). Striatal dopamine (d) and serotonin (e) levels were determined in mice administered with saline solution, mLPS, sLPS, and MPTP by HPLC. Data represent mean ± SEM (*n* = 5‐6) and were analyzed by two-way ANOVA, followed by a Tukey's *post hoc* test. Different letters indicate significant differences among the experimental groups (*P* < 0.05). Determination of MAO-B activity in the substantia nigra of mice administered with saline solution, treated with mLPS and sLPS (f). Data represent mean ± SEM (*n* = 7‐8) and were analyzed by Kruskal-Wallis, followed by Dunn's test. Different letters indicate significant differences among the experimental groups (*P* < 0.05).

The presence of motor symptoms characterizes Parkinson's disease due to dopamine depletion after the neuronal loss of dopaminergic neurons, mainly in the substantia nigra [1]. To explore the possible effect of systemic LPS stimulation on motor impairment, we evaluated motor coordination and forelimb strength. Motor coordination was only affected in mice groups exposed to MPTP (Figure 2(a)). Mice showed less motor coordination and forelimb strength since they presented lower scores than the control groups (Figure 2(a)). Interestingly, all groups exposed to MPTP also displayed less success in reaching the end of the support bar (*P* = 0.0457; Figure 2(b)).

Depressive disorders are frequent neuropsychiatric complications present in Parkinson's disease [37]. Also, systemic inflammation secondary to LPS exposure has been documented to induce depressive symptoms in mice [38, 39]. To explore the impact of systemic LPS exposure on depressive behavior in our model, we employed the sucrose preference test as an indicator of anhedonia. mLPS and MPTP groups showed significantly less sucrose consumption than the saline group (*P* = 0.0021 and *P* = 0.0002, respectively; Figure 2(c)). The sLPS and mLPS groups exposed to MPTP showed less preference for sucrose consumption when compared to the control group (sLPS (*P* < 0.0001), mLPS (*P* < 0.0001)). mLPS displayed less sucrose preference than the sLPS group (*P* = 0.001). The sLPS group presented less sucrose preference than the MPTP group (*P* < 0.0001). MPTP exposure in the mLPS group also showed diminished sucrose preference when compared to the mLPS group (*P* = 0.0148; Figure 2(c)). Two-way ANOVA showed that the interaction of LPS and MPTP exposure was significant in sucrose preference (interaction: *P* < 0.0001, *F* = 13.19; LPS: *P* < 0.0001, *F* = 12.18; MPTP: *P* < 0.0001, *F* = 113.3). In conclusion, LPS exposure and MPTP coadministration led to an increased preference for water consumption over the 2% sugar solution, suggesting anhedonia (Figure 2(c)).

Since motor impairment and depressive disorders are secondary to neuronal loss in the MPTP model, we determined the levels of dopamine and serotonin in the striatum of the different experimental groups. The two-way ANOVA showed that administration of LPS did not modify the dopamine nor serotonin, whereas MPTP only modified the dopamine (*F* 1, 28 = 17.49; *P* = 0.0003). However, LPS and MPTP challenges presented a significant interaction for both neurotransmitters (serotonin (*F* 2, 28 = 9.02; *P* = 0.0009); dopamine (*F* 2, 28 = 5.997; *P* = 0.0068)). The administration of mLPS caused a significant reduction in dopamine when compared to the saline group (*P* = 0.014; Figure 2(d)). Also, MPTP exposure significantly decreased the levels of both neurotransmitters compared to the saline-treated group (dopamine (*P* = 0.0008), serotonin (*P* = 0.0441); Figures 2(d) and 2(e)). LPS and MPTP coadministration also led to dopamine reduction (sLPS (*P* = 0.0058); mLPS (*P* = 0.0132), although this decrease was not significantly different from the MPTP group (Figure 2(d)). Curiously, previous LPS exposure to MPTP challenge restored serotonin levels (Figure 2(e)), and mLPS presented significantly higher levels of serotonin than the MPTP-exposed group (*P* = 0.0406; Figure 2(e)).

To ensure that the mice can correctly metabolize the MPTP after LPS challenge (sLPS and mLPS), we measured the activity of the enzyme monoamine oxidase B (MAO-B), which is the primary enzyme in transforming the MPTP into 1-methyl-4-phenylpyridinium (MPP+), the neurotoxic metabolite that enters neurons via the dopamine transporter. The activity of this enzyme was similar in all groups, and only the mLPS group presented significantly increased MAO-B activity (*P* = 0.023; Figure 2(d)).

Our data show that indeed MPTP challenge induces a motor deficit and anhedonic behavior in all experimental groups exposed to the neurotoxin. Anhedonic symptoms worsen when exposed to LPS beforehand. These phenomena are likely to be secondary to the decrease in dopamine [37], which is involved in the reward system [40], since the experimental sLPS and mLPS groups with MPTP presented an increase in serotonin compared to the MPTP group.

### 3.2. Multiple Low Doses of LPS Induce Persistent Systemic Inflammation

NF*κ*B activation via the toll-like receptor 4 (TLR4) pathway by LPS administration is a well-known event and widely documented [41]. NF*κ*B nuclear translocation leads to the production of inflammatory cytokines such as TNF*α*, IL1*β*, and IL6. However, prolonged exposure to LPS can sometimes induce tolerance [42, 43]. To rule out the possible LPS-induced tolerance effect in our multiple low doses of LPS administration (mLPS) experimental group, we measured both pro- and anti-inflammatory cytokines in serum samples.

The two-way ANOVA showed that administration of LPS modified the serum levels of pro- and anti-inflammatory cytokines IL1*β* (*F* 2, 25 = 16.50; *P* = 0.0002), IL6 (*F* 2, 32 = 37.09; *P* < 0.0001), TNF*α* (*F* 2, 28 = 66.47; *P* < 0.0001), and TGF*β* (*F* 2, 37 = 45.28; *P* < 0.0001), while MPTP modified IL1*β* (*F* 1, 25 = 8.779; *P* = 0.0066), IL6 (*F* 1, 32 = 27.48; *P* < 0.0001), IFN*γ* (*F* 1, 22 = 9.409; *P* = 0.0056), TNF*α* (*F* 1, 28 = 91.2; *P* < 0.0001), IL10 (*F* 1, 41 = 102.9; *P* < 0.0001), and TGF*β* (*F* 1, 37 = 61.64; *P* < 0.0001). LPS challenge acted synergistically with MPTP administration in IL1*β* (*F* 2, 25 = 7.815; *P* = 0.0023), IL6 (*F* 2, 32 = 8.458; *P* = 0.0011), TNF*α* (*F* 2, 28 = 5.665; *P* = 0.0086), and TGF*β* (*F* 2, 37 = 84.95; *P* < 0.0001).

**Figure 3:**
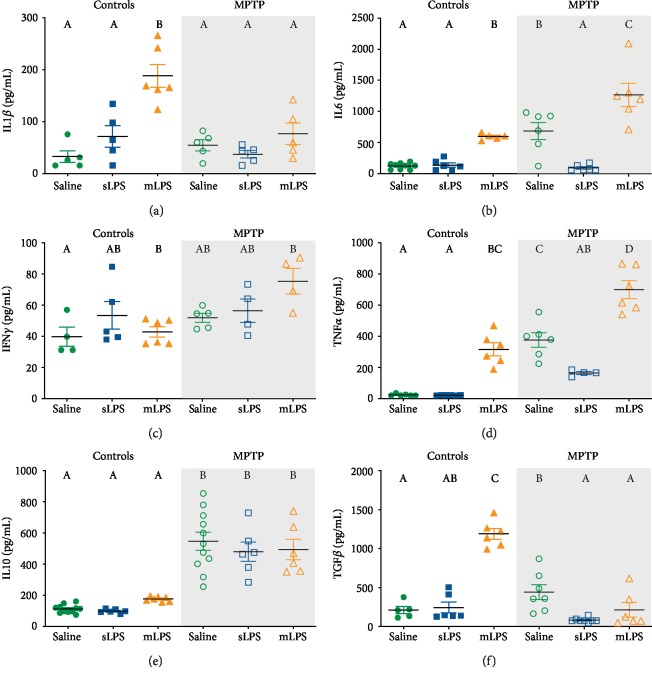
Serum inflammatory cytokines increase after LPS administration and MPTP challenge. Mice were administered with saline solution, mLPS (100 *μ*g/kg twice a week for three months), or sLPS (5 mg/kg), followed by MPTP (30 mg/kg for five consecutive days). The levels of IL1*β* (a), IL6 (b), IFN*γ* (c), TNF*α* (d), IL10 (e), and TGF*β* (f) were analyzed by ELISA. Data represent mean ± SEM (*n* = 5‐8) and were analyzed by two-way ANOVA, followed by a Tukey's *post hoc* test. Different letters indicate significant differences among the experimental groups (*P* < 0.05).

Multiple low doses of LPS (mLPS) increased the serum levels of IL1*β* (*P* < 0.0001), IL6 (*P* = 0.0094), TNF*α* (*P* < 0.0001), and TGF*β* (*P* < 0.0001) compared to saline, while the single LPS dose (sLPS) did not induce any changes in the cytokine levels (Figure 3). mLPS administration also raised IL1*β* (*P* = 0.0095), IL6 (*P* = 0.0258), TNF*α* (*P* < 0.0001), and TGF*β* (*P* < 0.0001) compared to the sLPS group (Figures 2(a), 2(b), 2(d), and 2(f)). The administration of MPTP also elevated the levels of IL6 (*P* = 0.0007), TNF*α* (*P* < 0.0001), and IL10 (*P* < 0.0001) compared to vehicle (Figures 2(b), 2(d), and 2(e)). However, when the MPTP stimulus followed a previous challenge with mLPS, it induced a significantly higher response in TNF*α* (*P* < 0.0001) and IL6 (*P* = 0.0015), while TGF*β* was decreased (*P* = 0.0002) when compared with the group administered only with MPTP (Figures 2(b), 2(d), and 2(f)). The sLPS and MPTP coadministration behaved similarly to the MPTP group. Interestingly, sLPS in conjunction with MPTP significantly decreased IL6 (*P* = 0.0011), TNF*α* (*P* = 0.0103), and TGF*β* (*P* < 0.0001) when compared with the group administered with MPTP only (Figures 2(b), 2(d), and 2(f)). The MPTP challenge in the mice previously exposed to mLPS induced a more pronounced response in IL6 (*P* < 0.0001) and TNF*α* (*P* < 0.0001) than the MPTP coadministration with sLPS (Figures 3(b) and 3(d)).

These results show that indeed chronic exposure to LPS maintains increased serum levels of the inflammatory IL6 and TNF*α* cytokines.

### 3.3. Systemic Inflammation Exacerbates Neuroinflammation after MPTP Challenge

After chronic systemic LPS challenge, an increase in proinflammatory cytokine production was observed (Figure 3). Peripheral proinflammatory cytokines can signal the brain by active transport through the BBB, the choroid plexus, or by afferent nerves such as the vagus nerve [44]. These peripheral inflammatory signals stimulate innate immune brain cells like microglia and astrocytes to respond with the same proinflammatory cytokines [15, 44]. To confirm this, we also assessed the presence of brain proinflammatory cytokines.

Two-way ANOVA showed that LPS modified the brain levels of IL1*β* (*F* 2, 25 = 15.65; *P* < 0.0001), IL6 (*F* 2, 34 = 28.25; *P* < 0.0001), TNF*α* (*F* 2, 36 = 56.42; *P* < 0.0001), IFN*γ* (*F* 2, 26 = 8.474; *P* = 0.0015), and TGF*β* (*F*2, 30 = 9.515; *P* = 0.0006), while MPTP modified IL1*β* (*F* 1, 25 = 44.4; *P* < 0.0001), IL6 (*F* 1, 34 = 92.88; *P* < 0.0001), TNF*α* (*F* 1, 36 = 61.89; *P* < 0.0001), IL10 (*F* 1, 34 = 101.5; *P* < 0.0001), and TGF*β* (*F* 1, 30 = 69.44; *P* < 0.005). The interaction of LPS and MPTP exposure was significant for IL1*β* (*F* 2, 25 = 6.609; *P* = 0.0001), IL6 (*F* 2, 34 = 29.64; *P* < 0.0001), and TGF*β* (*F* 2, 30 = 5.729; *P* = 0.0078).

**Figure 4:**
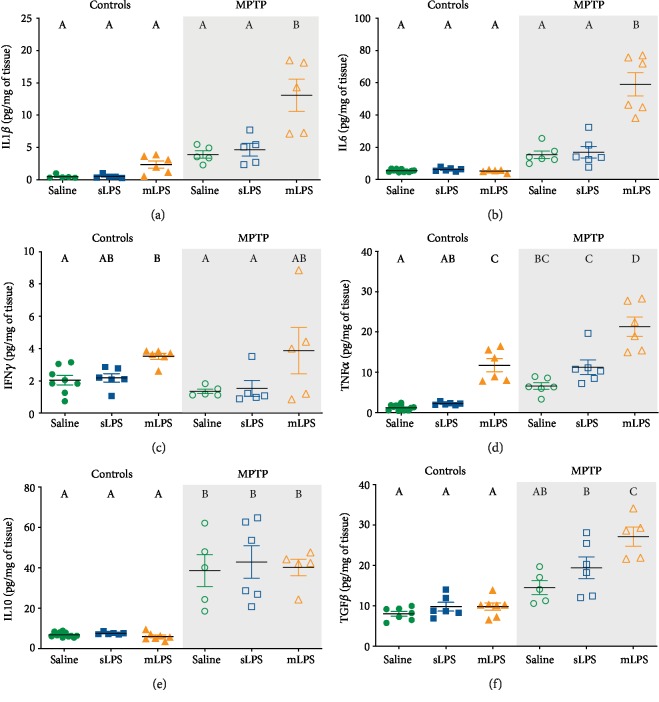
Brain inflammatory cytokines rise after LPS administration and MPTP challenge. Mice were administered with saline solution, mLPS (100 *μ*g/kg twice a week for three months), or sLPS (5 mg/kg), followed by MPTP (30 mg/kg for five consecutive days). The levels of IL1*β* (a), IL6 (b), IFN*γ* (c), TNF*α* (d), IL10 (e), and TGF*β* (f) were analyzed by ELISA. Data represent mean ± SEM (*n* = 5‐8) and were analyzed by two-way ANOVA, followed by a Tukey's *post hoc* test. Different letters indicate significant differences among the experimental groups (*P* < 0.05).

Mice administered with mLPS showed a significant rise in the brain levels of TNF*α* (*P* < 0.0001; Figure 3(d)) and IFN*γ* (*P* = 0.0158; Figure 4(c)), whereas MPTP administration induced a significant increase of TNF*α* (*P* = 0.0175; Figure 4(d)) and IL10 (*P* < 0.0001; Figure 4(e)). sLPS showed no significant changes in the brain cytokines measured compared to the saline group (Figure 4). The administration of mLPS in conjunction with the MPTP challenge led to higher levels of IL1*β* (*P* < 0.0001), IL6 (*P* < 0.0001), TNF*α* (*P* < 0.0001), and TGF*β* (*P* = 0.0003) compared to the MPTP group (Figures 4(a), 4(b), 4(d), and 4(f)). This group also presented similar levels of IL10 to the MPTP group (Figure 4(e)). The mice exposed to sLPS and MPTP stimuli behaved similarly to the MPTP group (Figure 4). MPTP challenge in the sLPS group significantly increased TNF*α* (*P* < 0.0001), IL10 (*P* < 0.0001), and TGF*β* (*P* = 0.0002) compared to the saline group (Figures 4(d)–4(f)). The mLPS group treated with the MPTP proneurotoxin significantly raised the inflammatory cytokines IL1*β* (*P* < 0.0001), IL6 (*P* < 0.0001), and TNF*α* (*P* < 0.0001) as well as IL10 (*P* < 0.0001) and TGF*β* (*P* < 0.0001) compared to the saline group (Figures 4(a), 4(b), and 4(d)–(f)). The main differences between the sLPS and mLPS groups was the presence of higher levels of TNF*α* in the mLPS group (*P* = 0.0001; Figure 4(d)) and that the mLPS group rises significantly the levels of IL1*β* (*P* = 0.0003), IL6 (*P* < 0.0001), TNF*α* (*P* < 0.0001), and TGF*β* (*P* < 0.0001) after the MPTP challenge (Figures 4(a), 4(b), 4(d), and 4(f)).

High TNF*α* levels may lead to the activation and nuclear translocation of NF*κ*B [45]. NF*κ*B, in turn, favors the transcription of inflammatory cytokines such as TNF*α*, IL1*β*, and IL6, perpetuating the inflammatory process [45]. We determined brain NF*κ*B p105 levels to show if proinflammatory cytokine production is related to NF*κ*B expression. The two-way ANOVA showed that administration of LPS (*F* 2, 16 = 4.743; *P* = 0.0241) and MPTP (*F* 1, 16 = 16.82; *P* = 0.0008) modified the NF*κ*B expression. No significant changes were observed in mice administered only with sLPS, mLPS, or MPTP (Supplementary [Supplementary-material supplementary-material-1]) compared to the saline group, though the levels of NF*κ*B p105 were significantly increased in the groups previously administered with sLPS or mLPS and then challenged with MPTP (*P* = 0.0317 and *P* = 0.0029, respectively; Supplementary [Supplementary-material supplementary-material-1]). Interestingly, all MPTP-treated groups behaved similarly (Supplementary [Supplementary-material supplementary-material-1]), and only the MPTP and mLPS-cotreated group was significantly different from mLPS (*P* = 0.0371; Supplementary [Supplementary-material supplementary-material-1]). It would have been more appropriate to evaluate the expression of phosphorylated NF*κ*B or its nuclear translocation since the increase in NF*κ*B p105 only indicates that there is more precursor for NF*κ*B p50 [46], which could be then translocated to the nucleus. However, a dual role for p105 has been established [47], and it could be possible that its increase could also indicate an attempt to regulate/inhibit the NF*κ*B pathway. This last hypothesis can be ruled out, since indirectly we observed a rise in IL1*β*, IL6, and TNF*α*, suggesting an active translocation of NF*κ*B to the nucleus, since the latter is responsible for the transcription of these molecules.

Single peripheral LPS challenge leads to BBB leakage [48], MPTP administration favors BBB dysfunction, probably secondary to the effect of TNF*α* [49, 50]. To evaluate if the systemic inflammation had any effect on the BBB, albumin coupled to FITC was administered intravenously 24 h before sacrifice. Striatal sections from control and sLPS mice did not show any presence of albumin (Supplementary [Supplementary-material supplementary-material-1]). Nevertheless, the mLPS group showed significantly higher levels of albumin compared to the saline (*P* = 0.0064) and sLPS groups (*P* = 0.0066; Supplementary [Supplementary-material supplementary-material-1]). These results provide evidence that chronic systemic inflammation by itself affects BBB integrity.

Since chronic systemic inflammation compromises BBB integrity, we next evaluated whether the changes observed in brain cytokines were the consequence of the systemic inflammation and not secondary to the entrance of LPS to the brain. To this end, LPS coupled to FITC was injected in the same mLPS scheme to explore this possibility (Supplementary [Supplementary-material supplementary-material-1]). The brain showed no FITC-LPS fluorescence (Supplementary [Supplementary-material supplementary-material-1]), reinforcing our observation that the neuroinflammatory effect is secondary to the systemic inflammation.

Our results show that chronic exposure to LPS induces persistent systemic and brain TNF*α*, which comprises the BBB integrity. Despite BBB leakage, circulating LPS does not enter the brain, suggesting its stimulation is in the periphery, and the cytokine synthesis afterward is secondary to circulating TNF*α*.

### 3.4. MPTP Intoxication after Systemic Inflammation Increases Iba1 Staining

Our results show that chronic stimulation with LPS does increase the expression of TNF*α* (Figure 4); in fact, the MPTP challenge after chronic exposure to LPS significantly raised the synthesis of IL1*β*, IL6, and TNF*α*. Since microglial cells respond to peripheral inflammatory signals by producing more inflammatory molecules such as TNF*α* [15, 44], we evaluated the morphology of these in substantia nigra and striatum, the two main areas that show dopamine depletion in Parkinson's disease [35].

**Figure 5:**
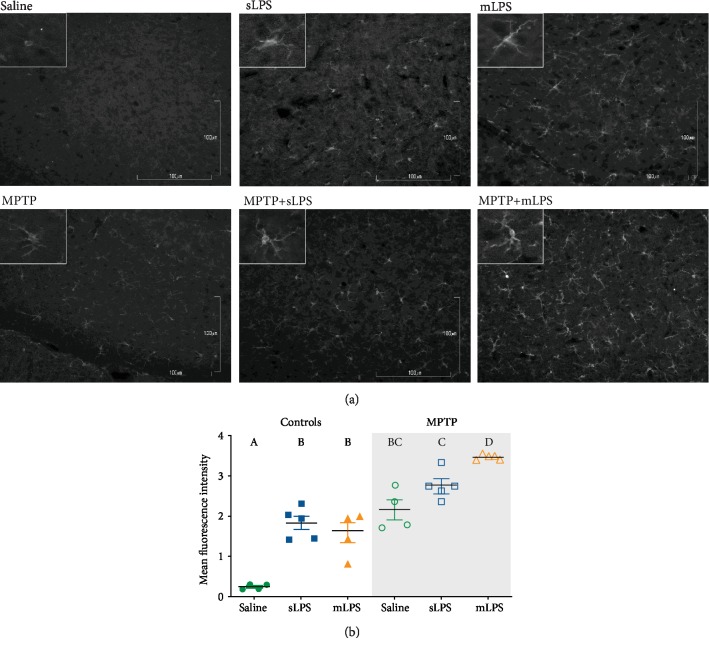
Iba1 staining increases in the substantia nigra after LPS administration and MPTP challenge. Representative images showing Iba1 immunodetection in grayscale in the substantia nigra of mice administered with saline solution, mLPS (100 *μ*g/kg twice a week for three months), sLPS (5 mg/kg), and MPTP (30 mg/kg for five consecutive days) (a). Quantification of the fluorescence intensity of Iba1 staining in the substantia nigra from the different experimental groups (b). Data represent mean ± SEM (*n* = 4‐6) and were analyzed by two-way ANOVA, followed by a Tukey's *post hoc* test. Different letters indicate significant differences among the experimental groups (*P* < 0.05).

Iba1, a microglial marker, was modified by LPS (*F* 2, 21 = 34.84; *P* < 0.0001) and MPTP (*F* 1, 21 = 134; *P* < 0.0001) in the substantia nigra as shown by the two-way ANOVA. LPS and MPTP showed a positive interaction in increasing Iba1 staining (*F* 2, 21 = 5.785; *P* = 0.01). This marker was significantly higher in the substantia nigra in all experimental groups when compared with the saline group (*P* ≤ 0.0002; Figures 5(a) and 5(b)). Interestingly, the MPTP group previously exposed to mLPS had significantly higher levels of Iba1 compared to the group treated with MPTP alone (*P* = 0.0002; Figure 5(b)). MPTP challenge in both LPS groups increased significantly Iba1 staining (mLPS (*P* < 0.0001), sLPS (*P* = 0.0044)) when compared to their respective control groups (Figures 5(a) and 5(b)).

**Figure 6:**
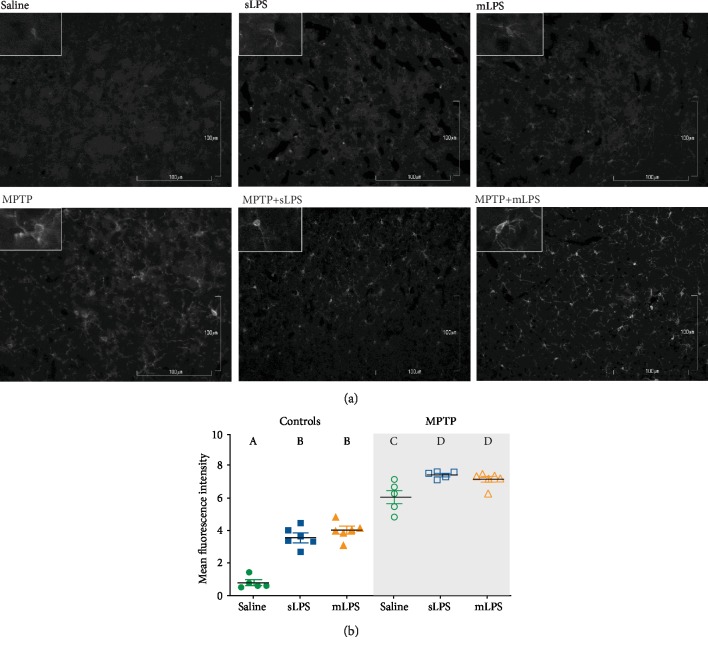
Iba1 staining increases in the striatum after LPS administration and MPTP challenge. Representative images showing Iba1 immunodetection in grayscale in the striatum of mice administered with saline solution, mLPS (100 *μ*g/kg twice a week for three months), sLPS (5 mg/kg), and MPTP (30 mg/kg for five consecutive days) (a). Quantification of the fluorescence intensity of Iba1 staining in the striatum from the different experimental groups (b). Data represent mean ± SEM (*n* = 4‐6) and were analyzed by two-way ANOVA, followed by a Tukey's *post hoc* test. Different letters indicate significant differences among the experimental groups (*P* < 0.05).

In the striatum, Iba1 staining showed a similar profile to that in the substantia nigra (Figures 6(a) and 6(b)). The two-way ANOVA showed that administration of LPS modified Iba1 staining in the striatum (*F* 2, 27 = 47; *P* < 0.0001) as well as MPTP (*F* 1, 27 = 408.7; *P* < 0.0001). Also, the two-way ANOVA showed a significant LPS and MPTP interaction (*F* 2, 27 = 9.422; *P* = 0.0008). The mLPS and sLPS groups have a similar Iba1 staining (Figures 6(a) and 6(b)), and MPTP exposure increased Iba1 when compared to saline, sLPS, and mLPS (all with a *P* < 0.0001; Figures 6(a) and 6(b)). MPTP treatment in the previously exposed LPS groups significantly increased Iba1 compared to the MPTP group (mLPS (*P* = 0.0404), sLPS (*P* = 0.0107); Figures 6(a) and 6(b)).

To further discern the role of microglia, minocycline was administered during the same three months as mLPS. Inhibition of microglia M1 polarization, the classically activated microglia with proinflammatory functions, reduced significantly IL1*β* and TNF*α* brain levels when challenged with MPTP and MPTP coadministered with mLPS almost to physiological levels (Supplementary [Supplementary-material supplementary-material-1]).

**Figure 7:**
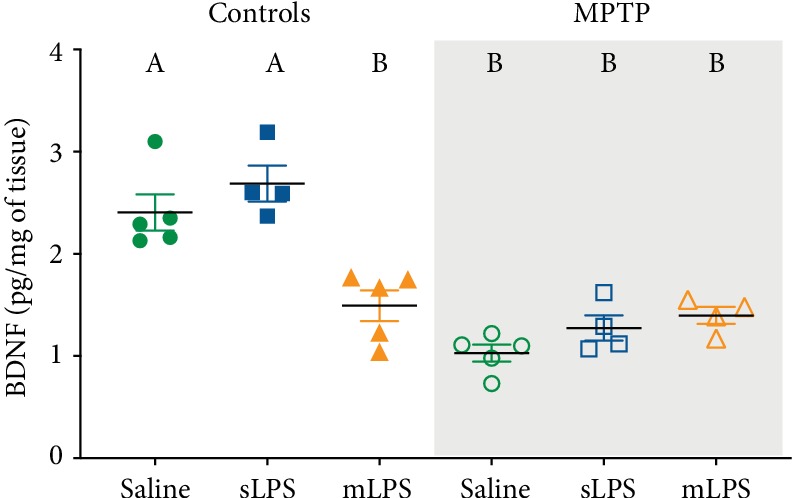
After LPS and MPTP challenges, brain BDNF levels are reduced. Mice were administered with saline solution, mLPS (100 *μ*g/kg twice a week for three months), sLPS (5 mg/kg), and MPTP (30 mg/kg for five consecutive days). The levels of BDNF were analyzed by ELISA. Data represent mean ± SEM (*n* = 4‐6) and were analyzed by two-way ANOVA, followed by a Tukey's *post hoc* test. Different letters indicate significant differences among the experimental groups (*P* < 0.05).

Additionally, we explored brain brain-derived neurotrophic factor (BDNF) levels. In the aging brain and under neuroinflammatory conditions, BDNF levels are reduced secondary to the M1 polarization of microglia and proinflammatory astrocytes [51–54]. The two-way ANOVA showed that administration of LPS modified the brain BDNF levels (*F* 2, 21 = 6.85; *P* = 0.0051) as well as MPTP (*F* 1, 21 = 69.57; *P* < 0.0001). Interaction between MPTP and LPS administration was significant (*F* 2, 21 = 14.17; *P* = 0.0001). Chronic exposure to systemic inflammation reduced BDNF significantly when compared to saline (*P* = 0.001; Figure 6) and sLPS groups (*P* < 0.0001; Figure 7), while MPTP exposure in all experimental models decreased further BDNF (*P* < 0.001; Figure 7).

Our data support that systemic inflammation leads to microglia activation possibly favoring an inflammatory M1 profile with increased Iba1 staining accompanied by increased brain IL1*β*, IL6, and TNF*α* production with a BDNF reduction, probably secondary to NF*κ*B activation.

### 3.5. Systemic Inflammation Exacerbates Damage after MPTP Challenge

Cytokine and BDNF levels were evaluated in whole brain lysates. Nevertheless, after the measurement of these molecules, we decided it would be more precise to evaluate in the primary two affected brain structures (striatum and substantia nigra), since the administration of peripheral LPS can induce neurodegeneration in the substantia nigra secondary to the microglial synthesis of TNF*α* and ROS [15, 55]. To test whether this happens with chronic exposure to systemic LPS, we evaluated cell viability with the MTT assay, cleaved caspase-3, and lipid peroxidation as damage markers.

**Figure 8:**
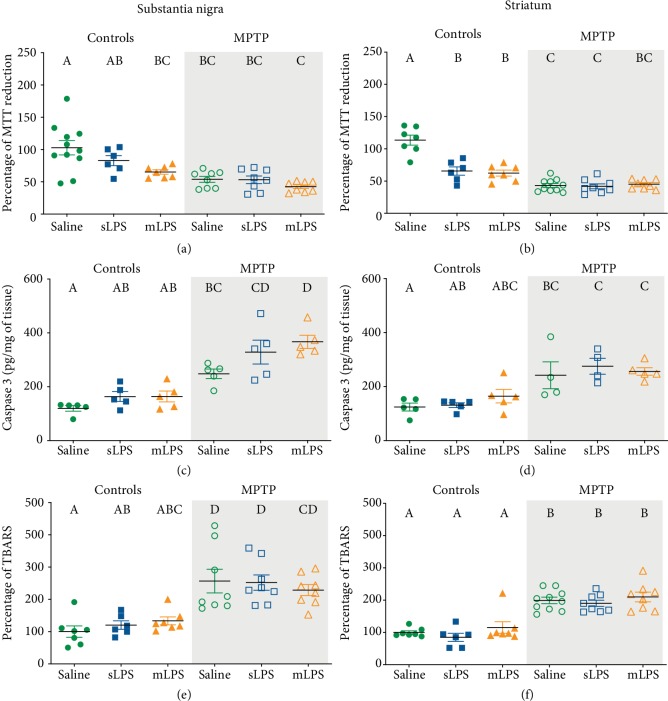
Cell viability decreases and lipid peroxidation and cleaved caspase-3 rise after LPS and MPTP challenges. Mice were administered with saline solution, mLPS (100 *μ*g/kg twice a week for three months), sLPS (5 mg/kg), and MPTP (30 mg/kg for five consecutive days). Cell viability assessed by MTT reduction (a, b), cleaved caspase-3 (c, d), and lipid peroxidation determined by TBARS method (e, f) were determined in the substantia nigra (a, c, e) and striatum (b, d, f). Data represent mean ± SEM (*n* = 4‐10) and were analyzed by two-way ANOVA, followed by a Tukey's *post hoc* test. Different letters indicate significant differences among the experimental groups (*P* < 0.05).

A decrease in cell viability determined by MTT reduction was observed in both substantia nigra and striatum of mice. In the substantia nigra, the two-way ANOVA showed that administration of LPS modified the cell viability (*F* 2, 42 = 5.339; *P* = 0.0086), as well as MPTP (*F* 1, 42 = 28.46; *P* < 0.0001). In the striatum, the two-way ANOVA also showed that administration of LPS modified the cell viability (*F* 2, 40 = 18.98; *P* < 0.0001), as well as MPTP (*F* 1, 40 = 92.32; *P* < 0.0001). Additionally, LPS and MPTP coadministration had a significant interaction in the striatum (*F* 2, 40 = 19.75; *P* < 0.0001). A reduction in cell viability in both substantia nigra and striatum was observed in mice treated with mLPS (*P* = 0.0099 and *P* < 0.0001, respectively; Figures 8(a) and 8(b)), whereas the sLPS dose only decreased cell viability in the striatum (*P* < 0.0001; Figure 8(b)). Similarly, MPTP reduced cell viability significantly when compared to the control (substantia nigra (*P* = 0.0002), striatum (*P* < 0.0001); Figures 8(a) and 8(b)). However, the previous exposure to LPS did not further diminish the reduction observed in the MPTP group (Figures 8(a) and 8(b)). Also, the sLPS-coadministered MPTP group presented significantly lower levels of MTT reduction in the striatum when compared to sLPS (*P* = 0.0182; Figure 8(b)).

The two-way ANOVA showed that administration of LPS modified the cleaved caspase-3 in the substantia nigra (*F* 2, 24 = 5.852; *P* = 0.0085), whereas MPTP modified the cleaved caspase-3 in the substantia nigra (*F* 1, 24 = 66.39; *P* < 0.0001) and the striatum (*F* 1, 22 = 34.03; *P* < 0.0001). MPTP exposure increased cleaved caspase-3 in the substantia nigra (*P* = 0.0142) and in the striatum (*P* = 0.0337) when compared to the saline control group (Figures 8(c) and 8(d)). The group previously administered with mLPS and subsequently with MPTP showed a significant increase in caspase-3 levels compared to the group administered only with MPTP (*P* = 0.0268); this effect was only observed in the substantia nigra of these mice (Figure 8(c)).

As an additional damage marker, we determined lipid peroxidation assessed by thiobarbituric acid reactive substances (TBARS) in the substantia nigra and the striatum (Figures 8(e) and 8(f)). The two-way ANOVA showed that administration of LPS did not modify the lipid peroxidation in the substantia nigra nor striatum, whereas MPTP modified the TBARS (*F* 1, 38 = 45.92; *P* < 0.0001) in the substantia nigra and the striatum (*F* 1, 40 = 96.66; *P* < 0.0001). We observed an increase of lipid peroxidation in the substantia nigra in mice exposed to MPTP alone, sLPS, and mLPS with MPTP challenge (*P* = 0.0003, *P* = 0.0004, and *P* = 0.0036, respectively; Figures 8(e) and 8(f)) compared to the saline group. In the striatum, lipid peroxidation showed a similar pattern (all with a *P* ≤ 0.0001) when compared to the control group (Figures 8(e) and 8(f)). However, the sLPS group challenged with MPTP presented with significantly raised lipid peroxidation in the substantia nigra (*P* = 0.0045) and the striatum (*P* < 0.0001) when compared to its sLPS control (Figures 8(e) and 8(f)). The mLPS-coadministered with MPTP group presented the same levels of lipid peroxidation in the striatum as the MPTP group and was significantly different from the mLPS group (*P* < 0.0001; Figures 8(e) and 8(f)).

Our results show that indeed with chronic LPS exposure, cell viability decreased in the substantia nigra and the striatum and was further reduced after MPTP challenge; these observations are in accordance with the higher levels of the active form of caspase-3. On the other hand, lipid peroxidation only was observed after MPTP administration, indicating that probably oxidative stress is not involved in the synergistic action of LPS and MPTP.

## 4. Discussion

Neurodegenerative diseases are chronic inflammatory and oxidative processes associated with aging that lead to neuronal death. The role of systemic inflammation has been established in neuroinflammation, but the contribution of chronicity of these inflammatory processes in the development of PD requires additional studies for their understanding. To explore this possibility, we used two different models of systemic LPS administration that induce neuroinflammation with a subsequent proneurotoxin MPTP challenge.

The sLPS administration scheme induces a well-known neuroinflammatory process [15]; however, we found that mLPS is a more complete neuroinflammatory model that *per se* resembles the MPTP model with higher brain TNF*α* levels, increased Iba1 staining in the substantia nigra and striatum, and diminished brain BDNF and dopamine levels, as well reduced cell viability and striatal dopamine. Our results are in accordance with other LPS models [56] that also induce nigrostriatal neurodegeneration secondary to neuroinflammation and microglial activation after LPS administration in the striatum [26] and substantia nigra [57] and of a single or repeated (four doses) systemic exposure [15, 25].

Chronic stimulation with LPS increased serum proinflammatory cytokines (IL1*β*, IL6, and TNF*α*), TGF*β*, but not IL10 (Figure 2). Interestingly, when the animal is subsequently challenged with MPTP, IL10 raised, and IL1*β* and TGF*β* decreased (Figure 2). Prolonged stimulation of TLRs can lead to reduced synthesis of IL1*β* through the action of IL10 in macrophages [58]. This event is likely to be secondary to the IL10 negative regulation of NLRP3 of the inflammasome pathway, which, in conjunction with caspase-1, are responsible for converting pro-IL1*β* to mature and functional IL1*β* [59]. TGF*β* lowering in the mLPS group after MPTP administration might be the result of the action of elevated IFN*γ* and TNF*α*. TGF*β* reduction in the mLPS group after MPTP administration might be the result of the action of elevated IFN*γ* and TNF*α*. IFN*γ* via STAT1 and TNF*α* via NF*κ*B induce inhibitory Smad7 expression, an antagonist of TGF*β* signaling pathway, thus lowering TGF*β* secretion [60, 61].

Although it has been shown that the repeated administration of LPS causes innate immune tolerance that could induce tolerant microglia [62], our data show the opposite. The mLPS administration induces a significant increase of the inflammatory cytokines (IL1*β*, IL6, and TNF*α*) secondary to the constant stimulation with LPS of the peripheral innate immune system. However, when peripheral immune tolerance to LPS has been demonstrated, it has been in models that employ higher doses of LPS (e.g., 300 *μ*g/kg daily for four days versus 100 *μ*g/kg twice a week for three months) and do or do not receive a second stimulus in the brain or *ex vivo* [63–65]. This peripheral immune tolerance is mainly by the downregulation of TLR4 expression and upregulation of CD200-CD200R and anti-inflammatory cytokines after epigenetic reprogramming [63–65]. On the contrary, our data support that the systemic TNF*α* induced by mLPS leads to microglial priming, exacerbating the inflammatory response after a stimulus such as MPTP and potentiating the damage induced by the neurotoxin. Systemic TNF*α* is transported through the BBB by TNF*α* receptors, promoting the activation of microglial cells, releasing additional TNF*α* and other cytokines establishing chronic neuroinflammation [15].

Unsurprisingly, we found compromised BBB permeability in mice administered with mLPS (Supplementary [Supplementary-material supplementary-material-1]), since previous reports have shown the disruptive effects of LPS on the BBB [66, 67]. The evidence shows that LPS stimulates the cerebrovascular endothelium and surrounding cells via prostanoids and NO [68–70]. Nevertheless, we did not detect LPS presence in the CNS after its intravenous administration (Supplementary [Supplementary-material supplementary-material-1]). Despite this, endogenous LPS can cross the BBB and reach the CNS under physiological conditions [71]. On the other hand, Banks et al. [70, 72] showed that disruption of the BBB with repeated injections of LPS did not enhance LPS entry into the CNS, and the effects of peripherally administered LPS are likely mediated through LPS receptors located outside the BBB. Thus, the effect observed in the brain results from the chronic systemic inflammation induced by the LPS treatment and not by direct LPS microglial stimulation.

mLPS generated an increase of brain TNF*α* but not of IL10 and TGF*β*, which are both anti-inflammatory cytokines, suggesting an imbalance of the anti-inflammatory mechanisms which causes a chronic proinflammatory state. It would be interesting to evaluate in the mLPS mice the levels of Parkin, since microglia exposed to LPS or TNF*α* downregulate Parkin, an anti-inflammatory regulator, increasing the vulnerability of the nigrostriatal pathway to degeneration [29, 73, 74].

Microglia can respond to damage by expressing specific growth factors. After a lesion in the striatum where dopaminergic neurons are mainly affected, microglia respond by releasing BDNF and glial cell-derived neurotrophic factor, both neuroprotective factors [75]. However, IL1*β* suppresses the release of BDNF [76]. Furthermore, the presence of M1 polarized microglia, the proinflammatory microglial cell, is associated with low BDNF brain levels in an LPS-induced depression-like model [77]. These observations could explain the reduction of BDNF in mLPS mice, simulating the changes related to aging and probably conferring susceptibility to damage in the experimental MPTP-treated groups [78].

MAO-B is the primary enzyme responsible for transforming MPTP to MPP+ in glial cells, and MPP+ enters into the dopaminergic neurons via dopamine transporter [79, 80]. In our models, MAO-B was not affected by the administration of LPS since the activity of MAO-B was not altered (Figure 2(f)), suggesting that astrocytes correctly metabolized MPTP and ensuring that the results observed are not due to a failure in the metabolism of the MPTP toxin. Moreover, our data show that mLPS increases MAO-B activity. MAO-B activity can be upregulated by p38 mitogen-activated protein kinases (p38 MAPK) in activated glial cells, microglia, and astrocytes [81, 82], and its overexpression in astrocytes causes dopaminergic neurodegeneration [83]. It is likely that in the mLPS model, the increase of MAO-B activity secondary to the p38 MAPK pathway, which plays a role in PD pathogenesis [84], could increase the amount of MPP+ generated, effectively increasing the neurotoxin dose.

The sLPS-coadministered MPTP model behaved very similar to the MPTP model presenting a similar cytokine and damage profile even though the sLPS increased the expression of Iba1. It is plausible that prior exposure to an acute inflammatory stimulus generates some protection or tolerance to the second proinflammatory stimulus, in this case, MPTP exposure, so the damage observed is mainly secondary to the neurotoxin. Nevertheless, MPTP challenge after mLPS administration led to significantly higher levels of IL1*β*, IL6, and TNF*α* than the other MPTP-treated groups, probably as a consequence of microglial priming since Iba1 staining was elevated in the substantia nigra when compared to MPTP- and sLPS-coadministered MPTP groups (Figures 4 and 5). Monocyte chemoattractant protein-1 (MCP-1/CCL2) secreted by astrocytes plays a role in microglia recruitment in the MPTP model [85], but it also increases after systemic LPS stimulation [42, 44]. It is probable that secreted MCP-1 favored microglial recruitment to the substantia nigra and striatum, thus increasing Iba1 staining in these structures (Figures 4 and 5). During aging, microglia generate higher levels of IL6, TNF*α*, and IL1*β* [86], possibly remaining in a chronic inflammatory state, similar to our model of mLPS.

Interestingly, mLPS also significantly raised IFN*γ* in the brain, which enhances the phagocytic activity of microglia [87] and induces the expression of MHC class II and costimulatory molecules, probably polarizing towards an M1 profile, therefore promoting persistent neuroinflammation with increased brain TNF*α* levels. With the administration of minocycline, an inhibitor of microglial M1 polarization, brain TNF*α* and IL1*β* levels were equal to those of the control group (Supplementary [Supplementary-material supplementary-material-1]). Even the increase of TNF*α* observed after mLPS and MPTP administration lowered to levels similar to control mice without minocycline treatment, indicating that microglial source of TNF*α* may play a crucial role in the development of neurodegenerative diseases. Deficiency of TNF*α* receptors in mice suppressed microglial activation and modified the brain susceptibility to MPTP damage, further supporting the role of microglia in TNF*α* production and participation in neuronal degeneration [88].

LPS exposure generates an inflammatory state very similar to that observed in depressive patients [89], and many studies claim that this inflammation, given by high levels of TNF*α*, IL6, and IL1*β*, can impact on serotonin metabolism [90]. Since proinflammatory cytokines can lead to the overactivation of the enzyme indoleamine-2,3-dioxygenase, which uses tryptophan as a substrate for the formation of kynurenine, tryptophan lowers, causing a decrease in serotonin synthesis [90]. The serotonin transporter is responsible for the recapture of serotonin; its overregulation caused by inflammation can also lead to a decrease in serotonin levels [91]. These observations are congruent with our results, where mLPS decreases serotonin inducing less sucrose preference similar to the MPTP model. Like in serotonin, cytokines have been shown to influence both the synthesis and the recapture of dopamine [92]. Mice from the different groups exposed to MPTP showed a decrease in motor coordination and forelimb strength when they were subjected to the balance bar test (Figures 2(a) and 2(b)), probably secondary to dopamine depletion comparable to other studies [29, 36, 93].

During PD, bradykinesia, rigidity, and resting tremor are the most common symptoms secondary to the depletion of dopamine levels [94, 95]. Notwithstanding, PD also presents nonmotor symptoms, including cognitive and psychiatric abnormalities, like dementia, depression, apathy, anxiety, and hallucinations [94]. Depression presents symptoms like anhedonia, the inability to perceive pleasure. Both our models previously exposed to LPS (mLPS and sLPS) and subsequently challenged with MPTP presented diminished preference in sucrose consumption, suggesting anhedonia (Figure 2(c)). This behavior arises from a deterioration of noradrenergic/serotonergic function that occurs secondarily to systemic inflammation. Evidence shows that brain and cerebrospinal fluid levels of norepinephrine [96] and serotonin in PD patients are decreased compared to healthy persons [97]. Systemic inflammation evoked by LPS could be generating a neuronal loss in multiple brain areas such as serotonergic neurons in the raphe nuclei, noradrenergic neurons in the locus coeruleus, dopaminergic neurons in the substantia nigra, and cortical neurons in regions interconnected with limbic structures, causing a depressive behavior in these mice similar to PD development [37]. We show that MPTP, in combination with LPS exposure, generated reduced cell viability, inflammation, and progressive neural degeneration in an *in vivo* model, suggesting MPTP and LPS cointeraction. MPTP and LPS interaction favors the NADPH oxidase-mediated release of superoxide free radicals in neuron-glia cultures; the pharmacological inhibition and genetic inactivation of NADPH oxidase prevented superoxide production and synergistic neurotoxicity [98]. MPTP and LPS have been proven to act synergistically [50, 98], stimulating microglia activation with inflammatory cytokine production and inducing neurotoxic A1 astrocytes [50]. Neurotoxic A1 astrocytes are present in the striatum of elderly [99], as well as in the substantia nigra and striatum of Parkinson's disease patient [100], and are considered to be coresponsible for neuronal loss since its blocking protects dopaminergic neurons in a Parkinson's disease murine model [101]. Further experiments on A1 astrocyte participation in these LPS models could be useful to elucidate more *in vivo* mechanisms that lead to dopaminergic neuron degeneration.

In conclusion, we demonstrated that chronicity in systemic inflammation generated by the repeated intraperitoneal administration of LPS induced elevated serum TNF*α* levels, leading to enhanced microglial neurotoxic response, subsequently increasing brain TNF*α* levels and raising MAO-B activity, consequently exacerbating MPTP damage. This chronic neuroinflammatory state results from the persistence of microglia activation and the subsequent production of proinflammatory cytokines, mainly TNF*α*. This model resembles more the impact of systemic inflammation in developing Parkinson's disease in humans than the model of the single LPS dose.

## Data Availability

All the data used to support the findings of this study are available from the corresponding author upon request.

## Supplementary Materials

Supplementary Materials Figure 1S: brain expression of NF*κ*B. (a) Representative Western blot bands for NF*κ*B p50 and p105 in the whole brain of mice challenged with saline solution, mLPS, sLPS, and MPTP. (b) Densitometric analysis of the expression levels of NF*κ*B p105. Figure 2S: increased permeability of the blood-brain barrier after chronic LPS administration. (a) Representative images showing the presence of labeled FITC-albumin in the striatum of mice treated with saline, sLPS, and mLPS. (b) Quantification of FITC-albumin present in the whole brain lysates obtained from the mice treated with saline, sLPS, and mLPS. Figure 3S: absence of LPS entry to the CNS. (a) Representative images showing the absence of labeled FITC-LPS in the striatum of mice treated with mLPS. (b) Quantification of FITC-LPS present in the total brain. Figure 4S: effect of minocycline administration on the levels of brain TNF*α* (a) and IL1*β* (b) in mice administered with saline, mLPS, and challenged with MPTP.
