# Supplementary material for: Chronic Systemic Inflammation Exacerbates Neurotoxicity in a Parkinson's Disease Model
Source: Oxid Med Cell Longev. 2020 Jan 11;2020:4807179. doi: 10.1155/2020/4807179 (PMC6982359; doi:10.1155/2020/4807179)
Supplement: Supplementary Materials — Figure 1S: brain expression of NFκB. (a) Representative Western blot bands for NFκB p50 and p105 in the whole brain of mice challenged with saline solution, mLPS, sLPS, and MPTP. (b) Densitometric analysis of the expression levels of NFκB p105. Figure 2S: increased permeability of the blood-brain barrier after chronic LPS administration. (a) Representative images showing the presence of labeled FITC-albumin in the striatum of mice treated with saline, sLPS, and mLPS. (b) Quantification of FITC-albumin present in the whole brain lysates obtained from the mice treated with saline, sLPS, and mLPS. Figure 3S: absence of LPS entry to the CNS. (a) Representative images showing the absence of labeled FITC-LPS in the striatum of mice treated with mLPS. (b) Quantification of FITC-LPS present in the total brain. Figure 4S: effect of minocycline administration on the levels of brain TNFα (a) and IL1β (b) in mice administered with saline, mLPS, and challenged with MPTP. [file 4807179.f1.pdf]

**Chronic systemic inflammation exacerbates neurotoxicity in a Parkinson's disease model**

Perla Ugalde-Muñiz[1], Ingrid Fetter-Pruneda[2], Luz Navarro[3], Esperanza García[4,†], Anahí Chavarría[1*]

[1]Unidad de Investigación en Medicina Experimental, Facultad de Medicina, Universidad Nacional Autónoma de México, CP06726, Mexico City, Mexico.

[2]Centro de Ciencias de la Complejidad, Universidad Nacional Autónoma de México, CP04510, Mexico City, Mexico.

[3]Departamento de Fisiología, Facultad de Medicina, Universidad Nacional Autónoma de México, CP04510, México City, Mexico.

[4]Laboratorio de Neuroinmunología, Instituto Nacional de Neurología y Neurocirugía Manuel Velasco Suárez, S.S., CP14269, Mexico City, Mexico.

† Deceased author.

*Corresponding author: Anahí Chavarría. anahi.chavarria@gmail.com.

## Supplementary materials

**Determination of brain NFκB** (Figure 1S)

Brain expression of NFκB was determined by Western blot in the whole brain of mice challenged with saline solution, mLPS, sLPS, and MPTP.

**Figure 1S:** Brain expression of NFκB. (a) Representative Western blot bands for NFκB p50 and p105 in the whole brain of mice challenged with saline solution, mLPS, sLPS, and MPTP. (b) Densitometric analysis of the expression levels of NFκB p105.

**Blood-brain barrier integrity** (Figures 2S)

Blood-brain barrier permeability was assessed by administrating albumin coupled to FITC intravenously in mice challenged with saline solution, mLPS and sLPS. Half of the brain was processed for immunofluorescent sections and the other half was processed to quantify albumin in brain lysates with a standard FITC-albumin curve.

**Figure 2S:** Increased permeability of the blood-brain barrier after chronic LPS administration. (a) Representative images showing the presence of labeled FITC-albumin in the striatum of mice treated with saline, sLPS, and mLPS. (b) Quantification of FITC-albumin present in the whole brain lysates obtained form the mice treated with saline, sLPS, and mLPS.

**LPS entry to the CNS** (Figures 3S)

The presence of LPS in the CNS was evaluated with the use of LPS coupled to FITC in mice challenged with saline solution, mLPS and sLPS. Half of the brain was processed for immunofluorescent sections and the other half was processed to quantify albumin in brain homogenates which were read on a fluorescent plate reader.

**Figure 3S:** Absence of LPS entry to the CNS. (a) Representative images showing the absence of labeled FITC-LPS in the striatum of mice treated with mLPS. (b) Quantification of FITC-LPS present in the total brain.

**Inhibition of microglia activation with minocycline** (Figures 4S)

To inhibit microglia activation minocycline was given orally in drinking water (10 mg/kg). Minocycline was administered during the same three months as mLPS administration. The animals were sacrificed three days after ending MPTP treatment to evaluate the brain TNFα and IL1ß levels by ELISA.

**Figure 4S:** Effect of minocycline administration on the levels of brain TNFα (a) and IL1ß (b) in mice administered with saline, mLPS, and challenged with MPTP.

## SUPPLEMENTARY MATERIAL

### Determination of NFκB by Western Blot

Whole mouse brains were homogenized with RIPA buffer and centrifuged at 10,000 rpm. Samples were normalized using the Lowry protein assay and prepared using the method of Laemmli. For GAPDH and NFκB p105/p50 detection, 20 µg of total protein was loaded per well on a denaturing polyacrylamide gel (SDS-PAGE). Gels were transferred to nitrocellulose membranes in a semi-humid chamber for 30 minutes using a Tris transfer buffer (400 mmol/mL Tris base, 70 mmol/mL glycine, 10% methanol). Membranes were incubated with the primary antibody for anti-NFκB p105/p50 (1:400; Abcam, ab7971) and anti-GAPDH goat anti-mouse (1:500; GeneTex, GTX89409) for 12 h, washed three times with TBS-tween 20 and then incubated with secondary antibodies coupled to fluorochromes [IRDye 800CW goat anti-rabbit (925-32211), and IRDye 680RD donkey anti-goat (925-68074) both from LI-COR]. The fluorescence intensity was measured using the Odyssey system of LI-COR, and the NFκB p105 signals were normalized to that obtained for GAPDH for its quantification and analysis. Three gels of three to four animals per group were analyzed.

(a)

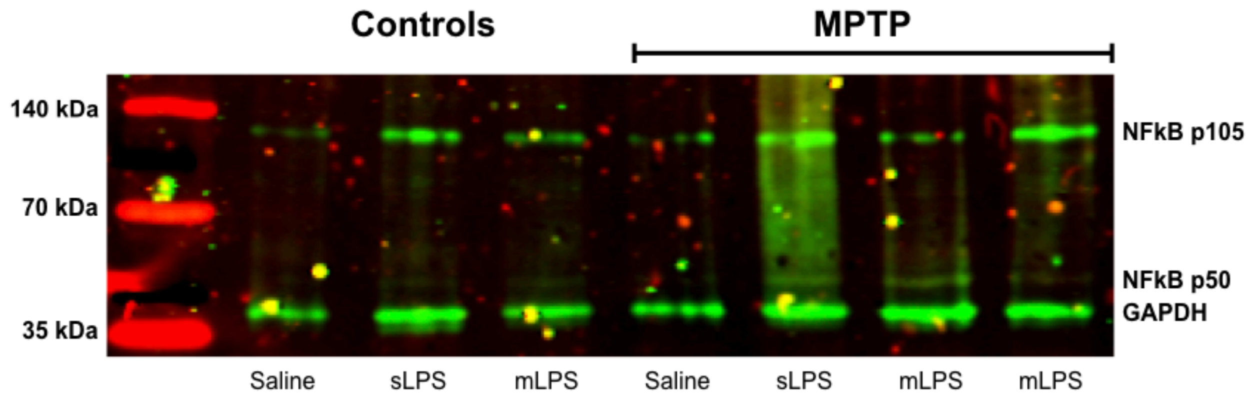

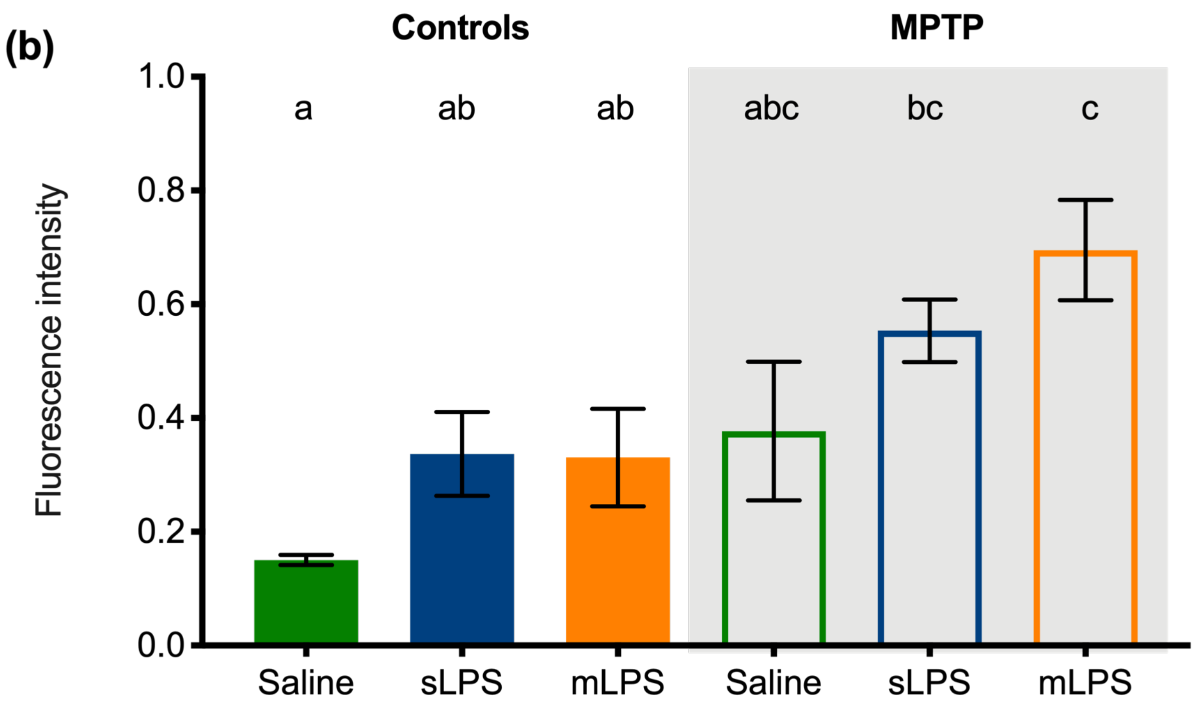


**Figure 1S:** Brain expression of NFκB. (a) Representative Western blot bands for NFκB p50 and p105 in the whole brain of mice challenged with saline solution, mLPS, sLPS, and MPTP. (b) Densitometric analysis of the expression levels of NFκB p105. The samples of the last two lanes correspond to the same mLPS with MPTP group. Data represent mean ± SEM (n = 3-4 mice per group) and were analyzed by two-way ANOVA, followed by a Tukey's *post hoc* test.

**Determination of blood-brain barrier integrity**

To establish the BBB permeability, fluorescein-coupled albumin (FITC-albumin; A9771, Sigma Chemical Co) was administered by intravenous administration in LPS treated groups (sLPS and mLPS; 10 mg/kg FITC-albumin) for three months as previously described [1]. Control mice received vehicle. Mice were sacrificed 24 h after last administration with sodium pentobarbital (50 mg/kg). The left half brain was fixed in 4% paraformaldehyde in phosphate-buffered saline solution (PBS), cryopreserved in 30% sucrose, and stored at −80 °C. Coronal sections (20 µm) of the striatum were obtained with a cryostat (Leica). The slices were adhered to silanized slides, nuclei were stained with a DAPI antifade solution, and observed with a fluorescent microscope Nikkon (Tokyo, Japan). The coordinates for the identification of the striatum (ML=1.5mm, AP=1.25mm, and DV= 3.0mm from Bregma) were based on the Paxinos Mouse Brain Atlas. The right half of the brain tissue was lysed in radioimmunoprecipitation assay (RIPA) buffer (10 mmol/L Tris, 140 mmol/L NaCl, 1% Triton X-100, 1% Na deoxycholate, 0.1% SDS, pH 7.5) containing a protease inhibitor, and centrifuged for 15 min at 10,000 rpm. Homogenates were read on a fluorescent plate reader at

488 nm excitation and 525 nm emission. A standard FITC-albumin curve was performed to evaluate the amount of FITC-albumin present in the brain lysates. Three animals per group were analyzed.

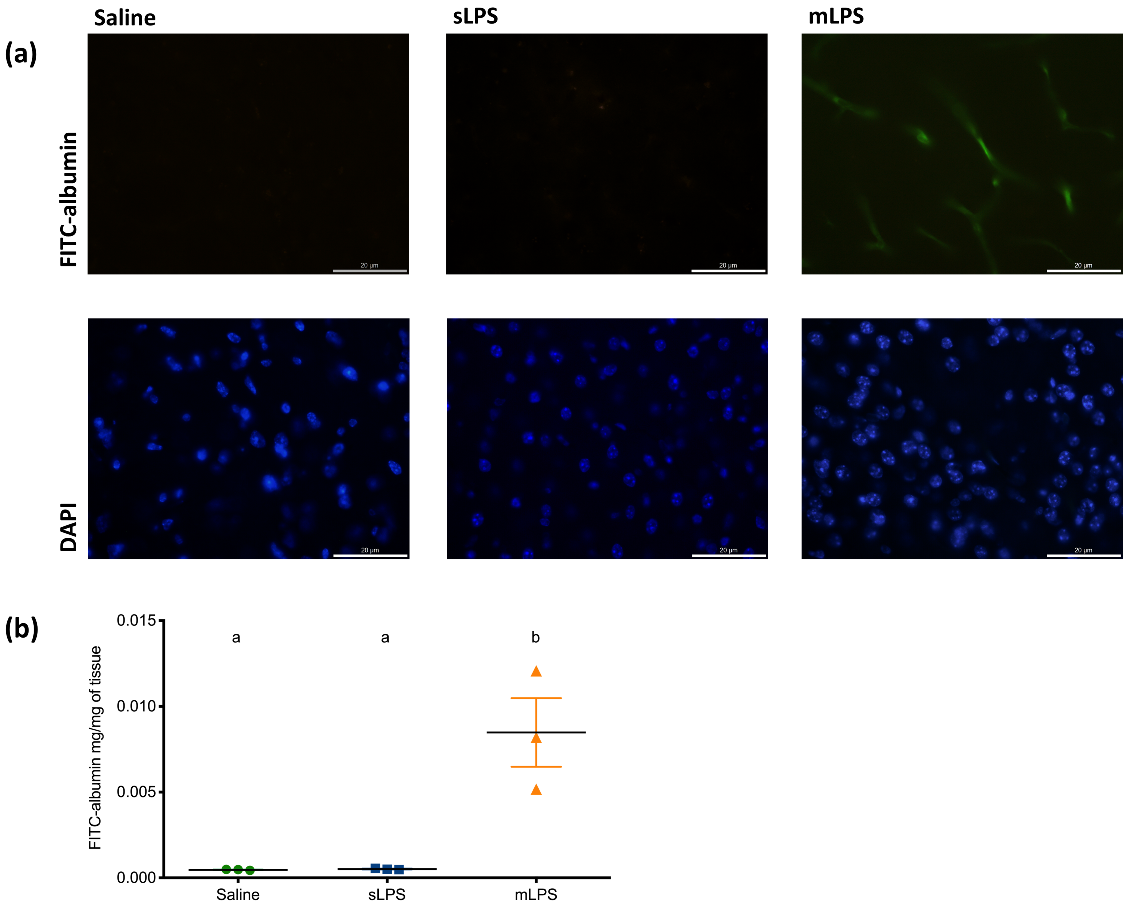


**Figure 2S:** Increased permeability of the blood-brain barrier after chronic LPS administration. (a) Representative images showing the presence of labeled FITC-albumin in the striatum of mice treated with saline, sLPS, and mLPS. (b) Quantification of FITC-albumin present in the whole brain lysates obtained form the mice treated with saline, sLPS, and mLPS. Data represent mean ± SEM and were analyzed by one-way ANOVA, followed by a Tukey's *post hoc* test. Data labeled with different letters indicate significant differences ($P < 0.05$).

## LPS entry to the CNS

The presence of LPS in the CNS was evaluated with the use of LPS coupled to FITC (F8666, Sigma Chemical Co). mLPS group was injected with 100 µg/kg FITC-LPS i.p. twice a week for three months. Mice were sacrificed 24 hrs after the last FITC-LPS administration. Control mice received vehicle. The left half brain was fixed in with 4% paraformaldehyde in PBS, cryopreserved in 30%

sucrose and stored at −80 °C. Coronal sections (20 μm) of the striatum were obtained with a cryostat (Leica). The slices were adhered to silanized slides, nuclei were stained with a DAPI antifade solution, and observed with a fluorescent microscope Nikkon (Tokyo, Japan). The coordinates for the identification of the striatum (ML=1.5mm, AP=1.25mm, and DV= 3.0mm from Bregma) were based on the Paxinos Mouse Brain Atlas.

The right brain tissue was lysed in RIPA buffer containing a protease inhibitor, and centrifuged for 15 min at 10,000 rpm. Homogenates were read on a fluorescent plate reader at 488 nm excitation and 525 nm emission.  At least three animals per group were used for statistical analysis. Student's t test was employed to compare control mice and FITC-LPS groups.

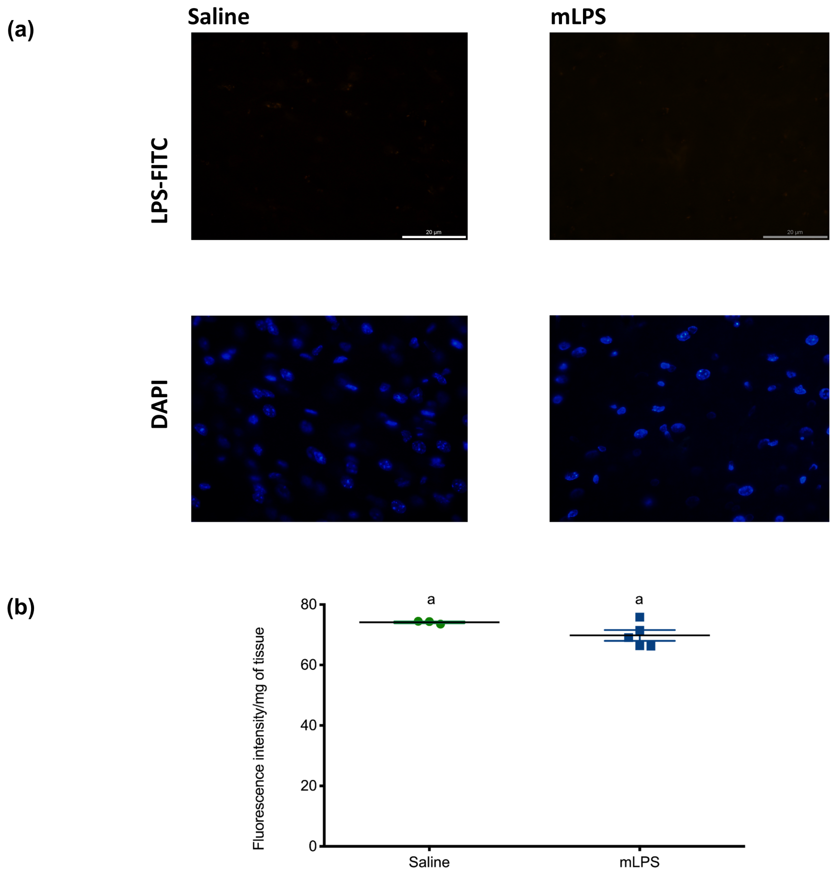

**Figure 3S:** Absence of LPS entry to the CNS. (a) Representative images showing the absence of labeled FITC-LPS in the striatum of mice treated with mLPS. (b) Quantification of FITC-LPS present in the total brain. Data represent mean ± SEM and were analyzed by Student's t. Data labeled with different letters indicate significant differences ($p < 0.05$).

**Inhibition of microglia activation with minocycline**

To inhibit microglia activation minocycline was given orally in drinking water (10 mg/kg). Minocycline was administered during the same three months as mLPS administration. The animals were sacrificed three days after ending MPTP treatment to evaluate the brain TNFα and IL1ß levels by ELISA.

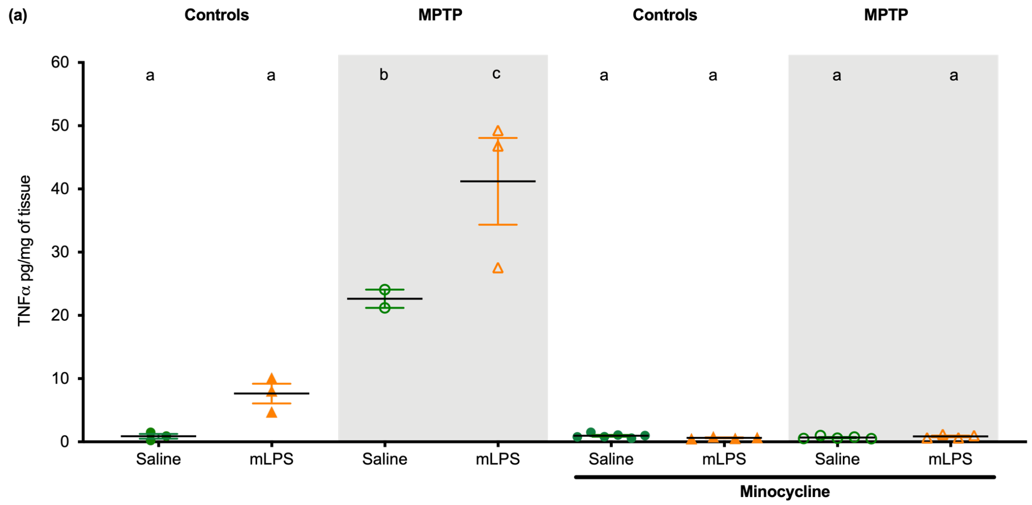


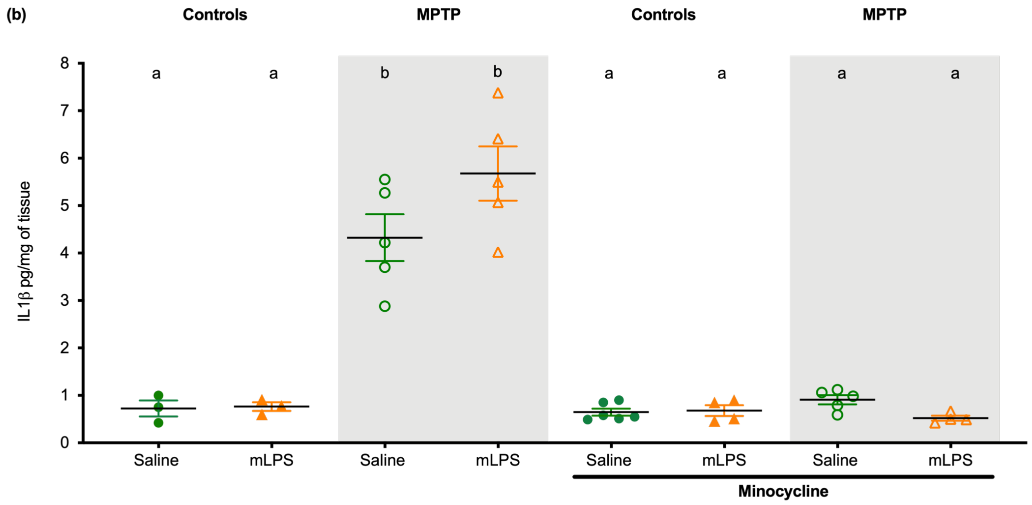


**Figure 4S:** Effect of minocycline administration on the levels of brain TNFα (a) and IL1ß (b) in mice administered with saline, mLPS, and challenged with MPTP. Data represent mean ± SEM and were analyzed by one-way ANOVA, followed by a Tukey's *post hoc* test. Data labeled with different letters indicate significant differences ($p < 0.05$).

## References

1. Suidan GL, McDole JR, Chen Y, Pirko I, Johnson AJ (2008) Induction of blood brain barrier tight junction protein alterations by CD8 T cells. PLoS One 3 (8):e3037. doi:10.1371/journal.pone.0003037
